# Deploying large language models for discourse studies: An exploration of automated analysis of media attitudes

**DOI:** 10.1371/journal.pone.0313932

**Published:** 2025-01-09

**Authors:** Qingyu Gao, Dezheng (William) Feng

**Affiliations:** Department of English and Communication, The Hong Kong Polytechnic University, Kowloon, Hong Kong SAR, China; Woldia University, ETHIOPIA

## Abstract

This study aims to provide an LLM (Large Language Model)-based method for the discourse analysis of media attitudes, and thereby investigate media attitudes towards China in a Hong Kong-based newspaper. Analysis of attitudes in large amounts of media data is crucial for understanding public opinions, market trends, social dynamics, etc. However, corpus-based approaches have traditionally focused on explicit linguistic expressions of attitudes, leaving implicit expressions unconsidered. To address this gap, the present study explored the possibility of using LLMs for the automated identification and classification of both explicit and implicit attitudes and evaluated the feasibility of implementing this approach on personal computers. The analysis was based on the framework proposed by Martin and White, which provides a structured approach for describing different aspects of media attitudes [1]. Meta’s open-source Llama2 (13b) was used for automated attitude analysis and was quantised for deployment on personal computers. The quantised LLM was used to analyse 40,000 expressions about China in a corpus of news reports from *Oriental Daily News*, a top-selling newspaper in Hong Kong. The results demonstrated that the quantised LLM can accurately capture both explicit and implicit attitudes, with a success rate of approximately 80%, comparable to that of proficient human coders. Challenges encountered during the implementation process and potential coping strategies were also discussed.

## Introduction

News media not only provide a wealth of information but also profoundly influence public opinions and behaviours through their attitudes and framings of events [2–4]. Analysing media attitudes can uncover biases inherent in different media sources and aid the public in reading media reports critically. However, achieving accurate analysis of media attitudes still heavily relies on manual annotation, which is both time-consuming and labour-intensive. Meanwhile, manual annotation necessitates a considerable number of annotators to make subjective judgements and annotations, rendering the process susceptible to individual biases and errors. The development of Natural Language Processing (NLP) has significantly facilitated text processing, enabling tasks such as sentiment analysis, topic modelling, and information extraction. Nevertheless, despite the high accuracy of NLP in automatically annotating language features with explicit forms, it still has limitations in effectively identifying and classifying more complex and less overt attitudes and emotions.

Recently, the advancement of Large Language Models (LLMs) has brought about a paradigm shift in NLP. By leveraging neural networks inspired by the human brain and trained on vast amounts of data, LLMs have achieved remarkable proficiency in “understanding” the human language. It follows that LLMs possess the potential to capture subtle semantic and contextual information, thus offering a promising avenue for analysing media attitudes. Against this backdrop, the present study sought to assess the ability of LLMs to capture attitudes embedded in texts. The objectives of this study include:

1. Assessing the ability of LLMs in capturing attitudes in language, including both explicit and implicit expressions.
2. Evaluating the feasibility and accuracy of LLM-assisted attitude analysis on local computers.
3. Conducting a preliminary LLM-assisted attitude analysis of the portrayal of China in the Hong Kong media, using the *Oriental Daily News* (*ODN*) as a case study.

The subsequent sections of this paper are organised as follows: Section 2 provides an overview of the Attitude system [1] and the development of attitude analysis methods, as well as the potential for annotation using LLMs. Section 3 provides a detailed description of our approach of utilising an LLM for attitude analysis. Section 4 presents the evaluation of the LLM practices. Section 5 centres on a discourse analysis regarding the *ODN*’s portrayal of China, based on the attitudes that the LLM identified. Finally, we summarise the limitations of our work and outline prospects for future research. The research highlights the feasibility of conducting LLM-based discourse analysis on personal computers, which facilitates efficient and secure data processing for researchers and practitioners without the need for extensive computational resources. Practical challenges encountered during the implementation process and proposed strategies to overcome these obstacles are also discussed.

## Background of study

### The Attitude system and attitude analysis (Fig 1)

For LLMs to perform effective attitude analysis, they must be furnished with a comprehensive and systematic framework for understanding attitudes, and the Attitude system meets this requirement perfectly. The system offers a nuanced taxonomy of attitudes that not only guides LLMs in identifying and classifying attitudes within texts but also paves the way for the automated analysis of media attitudes, enhancing the accuracy and efficiency of the process. The system comprises three major categories: Affect, Judgement, and Appreciation. Affect is about resources for construing emotional reactions, and it is further categorised into un/happiness, in/security, dis/satisfaction and dis/inclination. Un/happiness is to do with how much or to what extent we feel happy/unhappy. In/security deals with our anxious or assured feelings about the surroundings. Dis/satisfaction refers to our feelings of frustration and fulfilment relating to activities or states of events. Dis/inclination is to do with the desire for the condition of future events. Judgement is concerned with the assessment of human behaviour according to social sanction and social esteem. Judgement of social esteem involves the sub-categories of normality (how special someone is), capacity (how capable someone is) and tenacity (how resolute someone is). Judgement of social sanction is concerned with veracity (how truthful someone is) and propriety (how ethical someone is). Appreciation is the evaluation of things, which can be divided into reaction, composition, and valuation. Reaction refers to the degree to which things catch our attention. Composition is concerned with the internal structure of things, such as balance and complexity. Valuation is to do with the value of things, such as how original or authentic things are. The analytical framework allows for the systematic identification, categorisation, and quantification of media attitudes beyond the simple dichotomy of positive and negative.

**Fig 1:**
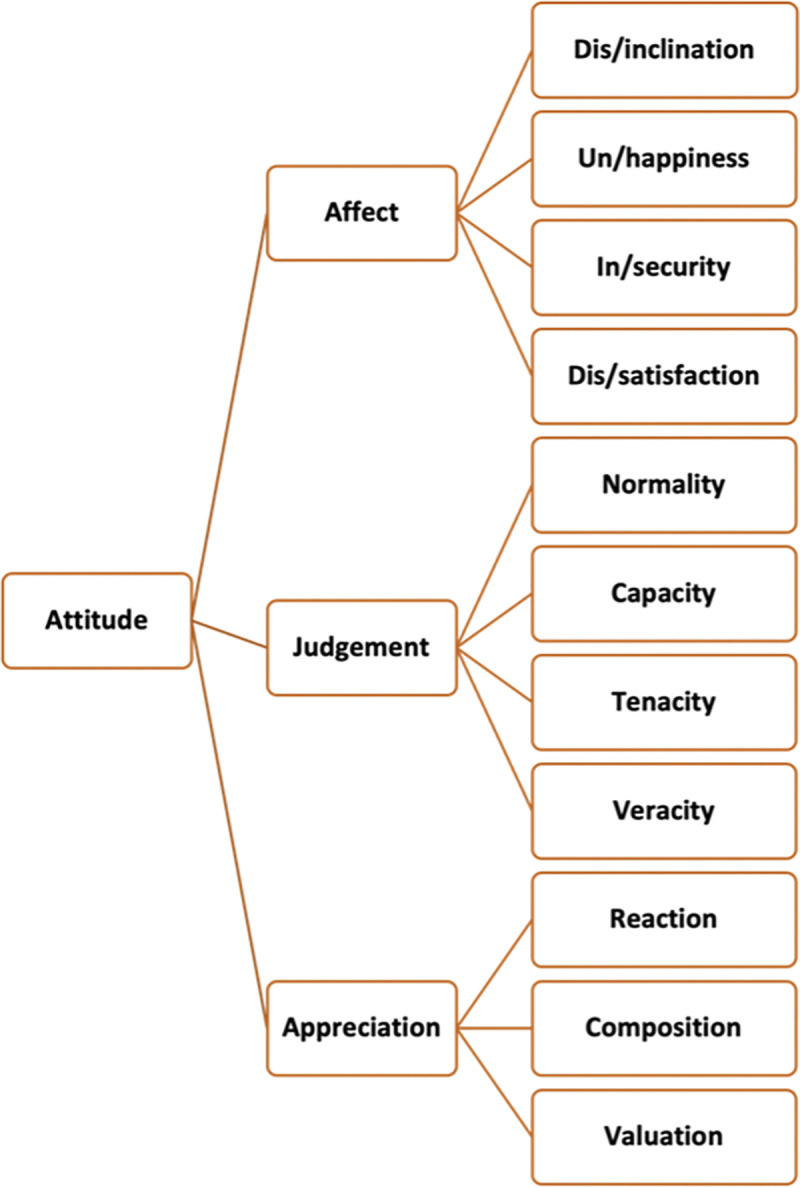
The Attitude system (Based on Martin & White, 2005 [1]).

Over the last two decades, the Attitude system has become a key framework for studying evaluation in discourse, with broad applications in applied linguistics, discourse analysis, and media studies. In the context of news reporting, editorials, and commentaries, the framework enables analysts to systematically map out how journalists and commentators employ language to convey their evaluations and stances on events, individuals, or phenomena. For example, researchers have used this system to investigate the expression of attitudes in news discourse, highlighting the system’s effectiveness in explicating the complexity of media attitudes [5,6].

In terms of analytical method, initially, the analysis typically involved qualitative examination of a small number of texts, with an emphasis on the “thick” description of them as well as the illustration of the framework. For instance, Martin and White analysed brief text examples from various types of texts, such as news reports, fictional narratives, and government documents, to specifically demonstrate the identification and interpretation of attitudinal resources within language [1]. Thompson (2008) took excerpts from short story collections to test the application of the attitude system, discussing three main issues encountered when applying the system and proposing suggestions for improvement [7].

With the need to identify patterns and trends of attitudes from a large amount of text, many researchers employed quantitative analysis. Bednarek utilised corpus linguistic methods to investigate evaluative language in newspapers through the quantitative calculation of its distribution and introduced a set of evaluative parameters for multidimensional analysis of evaluation [8]. Hunston emphasised the significant role of corpus linguistics in the study of evaluative language, which allowed researchers not only to identify the typical evaluative usage of specific words or phrases but also to quantify evaluative meanings across different text corpora and map meaning elements to formal elements through consistent patterns [9]. However, corpus-based approaches can only analyse explicit linguistic expressions of attitudes, leaving the implicit expressions unconsidered. To overcome this limitation, Fuoli introduced a method for annotating evaluative expressions in texts, which involved creating explicit annotation guidelines and continuously testing and refining these guidelines until maximum reliability was achieved [10,11]. The method aimed to control subjectivity in the annotation process, so as to enhance the transparency, reliability, and replicability of the analysis. However, manual annotation requires substantial human effort and is inevitably influenced by individuals’ subjective judgements. To further address these challenges, researchers have attempted to use automatic annotation of emotions and attitudes. For example, Taboada and Grieve proposed an automatic text evaluation method that determines sentiment orientation by identifying adjectives in the text and calculating their Pointwise Mutual Information (PMI) values [12]. Balahur et al. proposed to utilise a knowledgebase to construct the EmotiNet model, enabling automated sentiment detection in textual data [13]. Gao and Feng explored the detection of sentiment and its source and target in news texts by using semantic role relations and sentiment lexicons [14]. Loureiro et al. trained a deep learning model to ascertain the sentiment polarity of texts and subsequently fine-tuned the model to discern 11 distinct emotions [15,16]. Xu et al. employed techniques such as Bidirectional Encoder Representations from Transformers (i.e. BERT), Long Short-Term Memory, and Multi-Head Attention Mechanism to propose a deep learning model for automatic attitude annotation in dialogue texts [17]. To tackle the issue of standardisation, Read and Carroll proposed a set of machine-readable Appraisal annotation schemes for collective analysis, emphasising inter-annotator agreement [18]. Taboada and Carretero introduced a comprehensive framework for the annotation of evaluative expressions, facilitating the consistent identification and categorisation of evaluative language across corpora in different languages [19]. Through this approach, researchers can better understand and analyse the use and expression of evaluative language in various contexts.

Despite these remarkable achievements, automated annotation processes still fall short of human-level recognition when it comes to complex and implicit attitude expressions. The main challenge is that these methods primarily rely on explicit linguistic features and patterns, such as keywords and vocabulary frequencies, co-occurrence patterns, and syntactic structures to determine attitude. Consequently, these approaches struggle to accurately capture implicit attitudinal expressions and contextual information in language, posing a challenge for comprehending complex language expressions of attitudes. Furthermore, the scattered nature of attitudes in texts continues to pose challenges for current methods. The emergence of large language models promises the potential to break the current deadlock.

### The annotation potential of LLMs

LLMs are distributions of language probabilistic parameters, structured as multilayer neural networks, and trained on massive amounts of data. By mastering intricate language meanings and structures through these probability distributions, LLMs possess the ability to “understand” language. Compared to traditional NLP methods such as Support Vector Machines and Deep Learning, LLMs excel at capturing contextual information and semantic associations [20]. Therefore, they theoretically fulfil the fundamental requirements for attitude analysis.

LLMs have achieved satisfactory results in various NLP annotation tasks. For instance, Frei and Kramer, and Zhang et al. tested the performance of an LLM in Named Entity Recognition tasks separately, demonstrating their accurate extraction of domain-specific terms from texts [21,22]. Yu et al. explored the feasibility of using an LLM for apology annotation and found that, through optimised prompts, the model could identify key features of apologies with high accuracy [23]. Ostyakova et al. compared LLM annotation results with those of professional and non-professional annotators, revealing that the multi-step pipeline-processed LLM annotations achieved performance comparable to human annotators [24]. In another study by Gilardi et al., language annotations by ChatGPT (a type of LLM) exhibited higher accuracy than those by crowd workers, and the inter-annotator agreement surpassed that of human annotators [25]. This finding was further supported by Ding et al., who found that LLMs had the potential to accurately annotate data for various NLP tasks, with significantly less time and cost compared to human annotators [26].

While the aforementioned research demonstrated the excellent performance of LLMs in annotation tasks, the subtle and indirect nature of attitudes in discourse makes attitude annotation more challenging, compared to tasks with explicit external representations, such as Named Entity Recognition and apology behaviour. Additionally, Nedilko pointed out that common online conversational LLMs, like ChatGPT, may exhibit fluctuations in output and are constrained by the context window [27]. Xu et al. also raised concerns about security and privacy when utilising online LLMs [17]. Therefore, we emphasise the feasibility of deploying LLMs locally for automated attitude recognition to mitigate potential risks like data leakage and to evaluate the accuracy of automated attitude recognition.

### Methodology

To evaluate the feasibility and accuracy of conducting LLM-assisted attitude analysis on local computers, we selected the representation of China in the *ODN* as the analysis sample, which is from a larger project conducted by the authors, and demonstrated how to deploy LLM locally for automated attitude recognition. *ODN* is a leading Chinese-language newspaper in Hong Kong, and its attitudes towards China constitute an information-rich case for analysis. A total of 40,000 expressions from the *ODN* about China were randomly extracted from a large corpus of Hong Kong media’s expressions of China, amounting to 2.523 million words. In this section, we will provide a detailed description of our research methodology in terms of local deployment of the model, optimisation of prompts, and evaluation methods.

### Local deployment of the model

Considering availability and ease of deployment, we chose the Llama2 model for local deployment. Llama2 is the second-generation open-source model in the Llama series, developed by Meta, and was the premier model available at the commencement of the project. It has undergone pretraining on a high-quality dataset of approximately 2 trillion tokens, as well as iterations of internal structure and algorithms, and was considered the best open-source model [28]. Llama2 offers three versions with different parameter sizes: 7B, 13B, and 70B. The larger the parameter size, the higher the computational requirements for deploying the model. The 7B version requires 13 GB of RAM, the 13B version requires 24 GB, and the 70B version requires 140 GB of RAM. Even after 4-bit quantisation, the 70B version still requires approximately 40 GB of RAM. In our hardware environment with an i7-12700K processor and 32 GB of RAM, we chose the 13B version to maximise performance.

Quantisation is a method of converting the original 32-bit floating-point parameters of the model into lower precision to save RAM. To ensure that prompts and the text being analysed can be passed to the model simultaneously, we chose to quantise the Llama2-13B model to further compress the RAM space it occupies. After multiple tests, we found that the Q5KM quantisation method effectively saved RAM space while preserving the majority of the model’s performance. Finally, we set up a server running the quantised Llama2 13B model locally using the llama.cpp script.

### Prompt engineering

Prompts are the information provided by users through the Application Programming Interfaces (APIs) or direct interaction with LLMs. They contain instructions or questions to be passed to the model and may include context, input data, output instructions, or examples. Prompt engineering focuses on the development and optimisation of prompts to help users apply LLMs in various scenarios and improve their ability to handle complex tasks. In this study, we used the CRISPE framework proposed by Matt Nigh to construct prompts with complex structures for automated attitude recognition [29]. We adopted a progressive trial-and-error approach to optimise the prompts. In what follows, we will describe how we designed the prompts according to the five components of the CRISPE framework including Capacity and Role, Insight, Statement, Personality, and Experiment.

### Capacity and role

This section states the desired capabilities and role of LLMs. Based on the research requirements, our designed prompt was “You are a trained attitude analysis expert who can identify different types of attitudes from news texts.”

### Insight

This section provides background information and context. In this study, we provided the LLM with category features of the Attitude system as background information. In the initial attempts, we used the zero-shot approach [30] to directly pass the key elements of the 12 attitude categories to the LLM, such as “Attitudes are divided into 12 categories: Happiness, involving individuals’ feelings of joy or sadness; Security, involving individuals’ anxiety or reassurance about the environment”. However, the direct transmission of key elements did not yield satisfactory results. We believed that the key elements were too abstract for the LLM, leading to increased confusion. Therefore, we subsequently adopted the few-shot approach [31], providing an example of each attitude after giving the key elements to help the model “understand” the attitude more intuitively, such as “Composition, expressing the evaluator’s evaluation of whether the internal composition of something is reasonable or balanced; *When examining this painting*, *the artist appreciates the colours and lines*, *considering the balance and harmony between them add depth and aesthetics to the artwork*. This sentence expresses the attitude of Composition.”

### Statement

This section describes what we want the LLM to do. We need the model to identify attitudes in the discourse according to the Attitude system and evaluate their polarity. Therefore, we designed the prompt as “Please identify the attitudes from the given text based on the aforementioned attitude categories and determine whether the attitude is positive (Pos) or negative (Neg).”

### Personality

This section describes the desired manner of response from the LLM. To facilitate subsequent statistical analysis, responses from the LLM were requested in a coded format: “Please respond by listing the attitudes in the order of their occurrence, using a coded schema that pairs the name of the attitude with its polarity, for example, *Satisfaction-Pos* to denote positive satisfaction, and *Propriety-Neg* to indicate negative propriety.”

### Experiment

This section outlines additional requirements posed to the LLM: “A single text may encompass multiple attitudes; identify all of them. Begin by listing the codes for the attitudes, followed by a brief explanation if necessary. Note that providing an explanation is not mandatory.”

**Fig 2:**
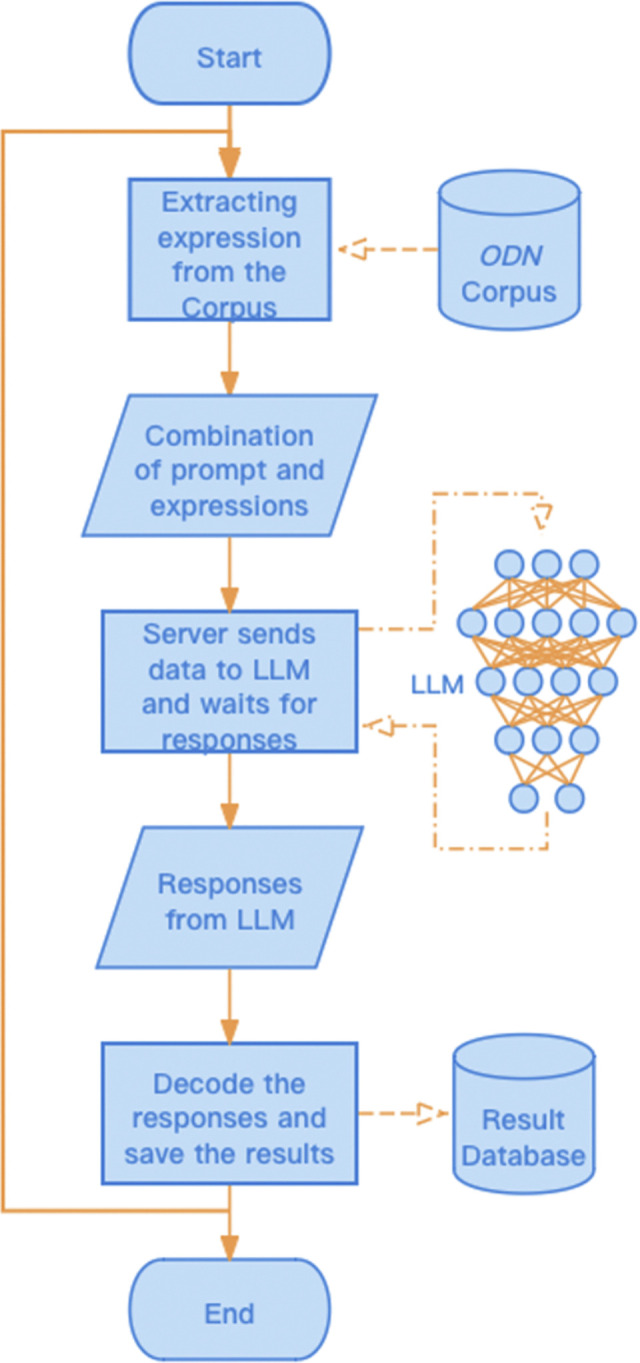
Workflow of the project.

The five components are integrated into a comprehensive Prompt, which is then sequentially paired with an expression drawn from a set of 40,000 expressions for processing by the model. The outcomes returned by the model, along with the corresponding expressions, are stored in a database for subsequent analysis. A diagram illustrating the complete workflow is provided in Fig 2.

### Evaluation methods

To evaluate the performance of the LLM in attitude analysis, we randomly sampled 915 expressions from the results. In the sampling process, we considered the representation of various attitude types and polarities, with a particular emphasis on balancing positive and negative within a diverse range of attitudes, to ensure a comprehensive and representative evaluation. The subset was independently annotated by two researchers. When discrepancies arose, a third researcher was consulted to reach a consensus. We then compared the manual annotations with the machine-annotated results. Only when both the attitude type and polarity matched the manual annotations were the machine-annotated result considered correct. We calculated the model’s accuracy, precision, recall, and both macro-average and micro-average F1 scores for a systematic assessment. Accuracy measures the proportion of correctly predicted samples out of the total number of samples. It includes both cases with attitudes (i.e., positive samples) which are correctly identified and cases with no attitudes (i.e., negative samples) which are correctly labelled as such. Precision measures the proportion of correctly predicted positive samples among all predicted positive ones, that is, the percentage of the correct attitudes identified by the LLM among all attitudes it identified. Recall is the proportion of the correct attitudes identified by the LLM to those which actually express the attitude as identified in manual coding. A low Precision indicates that among the samples where the LLM predicts an attitude, there are many that do not actually have the attitude, which can be referred to as overinterpretation. Conversely, a low Recall suggests that there are many instances where an attitude is present but is not predicted by the LLM, which can be termed as underinterpretation. Considering the large among of negative samples (i.e., cases without attitudes), the first metric of Accuracy is less revealing than the latter two. Therefore, the harmonic mean of Precision and Recall, that is, the F1 score, is a key metric for evaluating the performance of classification models. In multi-class classification, the macro-average F1 score focuses on the performance of individual classes and combines them with equal weight while the micro-average F1 score pays more attention to the performance of the overall dataset, considering the differences in sample sizes across various classes. The formulas are listed below:

$$Accuracy=\frac{TP+TF}{TP+FP+TN+FN}$$

$$Precision=\frac{TP}{TP+FP}$$

$$Recall=\frac{TP}{TP+FN}$$

$$F1=2∙\frac{Precision∙Recall}{Precision+Recall}$$

$${F1}_{macro-ave}=\frac{1}{n}\sum_{i=1}^{n}{F1}_{i}$$

$${F1}_{micro-ave}=\frac{\sum_{i=1}^{n}{Precision}_{i}\times {Recall}_{i}}{\sum_{i=1}^{n}\left(\frac{{Precision}_{i}\times {Recall}_{i}}{{Precision}_{i}+{Recall}_{i}}\right)}$$

*TP (True Positives)*: *The number of samples that the model correctly predicted as the positive class*.

*TN (True Negatives)*: *The number of samples that the model correctly predicted as the negative class*.

*FP (False Positives)*: *The number of samples that the model incorrectly predicted as the positive class*.

*FN (False Negatives)*: *The number of samples that the model incorrectly predicted as the negative class*.

### Evaluation of results

The deployed LLM successfully identified 38,215 attitudes from 40,000 expressions, and the evaluation result demonstrated an excellent performance in attitude analysis tasks. It significantly reduced the time required for attitude identification while achieving results comparable to manual recognition. The evaluation results are presented in Table 1 below. From the table, it is evident that the LLM exhibited high accuracy, and satisfactory precision and recall. The model demonstrated balanced and robust recognition across all attitude categories.

**Table 1:** Attitude analysis performance metrics (N=915).

Attitude Type	Accuracy	Precision	Recall	F1
Overall	0.95	0.81	0.80	0.80 (Micro)
				0.80 (Macro)
Happiness	0.95	0.68	0.92	0.78
Security	0.93	0.76	0.75	0.75
Satisfaction	0.95	0.88	0.77	0.82
Inclination	0.94	0.86	0.75	0.80
Normality	0.99	0.90	1.00	0.95
Capacity	0.92	0.78	0.68	0.73
Tenacity	0.95	0.88	0.79	0.83
Veracity	0.96	0.82	0.85	0.84
Propriety	0.93	0.86	0.70	0.77
Reaction	0.96	0.86	0.88	0.86
Composition	0.94	0.66	0.85	0.74
Valuation	0.94	0.74	0.79	0.76

From the perspective of Accuracy, the LLM demonstrated excellent performance across all attitude types, with a score of 0.95, indicating that the model accurately identified the correct attitude in the vast majority of cases. Notably, the Accuracy for Normality reached a peak of 0.99. We attribute the high accuracy in recognising Normality to two potential reasons: firstly, Normality often involves comparisons with widely accepted social standards, a common pattern in discourse, thus providing ample stimuli for the LLM during training. Secondly, Normality is frequently expressed through easily recognised vocabulary and expressions, which are more easily captured by the model. For instance, in Example 1, the explicit attitudinal term “normal” aided the LLM in identifying Normality. However, as explained in the previous section, this metric includes a large number of negative samples (i.e., cases with no attitudes which are correctly labelled as such) and thus is not adequate in evaluating the capacity of the LLM.

**Example 1**:

Jia Qinglin did not receive many votes, which is a normal phenomenon in political democracy.

(LLM marked as Normality-Pos)

Precision and Recall measure the model’s accuracy in terms of the attitudes it identified (Precision) and its ability to capture all attitude expressions (Recall), respectively. Our deployed LLM has achieved scores of approximately 0.8 for both, indicating that 80% of the identified attitudes are correct and that the model can identify 80% of attitudes from all samples. Considering the scattered and complex distribution of attitudes in discourse, this performance is commendable. However, the proportions are below 80% for half of the attitude types, revealing the LLM’s occasional overinterpretation and underinterpretation.

Overinterpretation, as manifested in comparatively lower figures of Precision, is mainly found in the analysis of Composition, Happiness, Valuation, Security, and Capacity in the research. The susceptibility of these attitude types to overinterpretation was likely due to the model’s heightened sensitivity to textual cues or its generalised comprehension of contextual information. Specifically, misjudgements in Composition may stem from the model’s misinterpretation of information synthesis; Happiness could be erroneously classified as a consequence of the model’s over-sensitive response to neutral or ambiguous emotional expressions; Valuation might be misconstrued due to an insufficient grasp of the nuances within particular contexts; Capacity may be misidentified because of the model’s tendency to overgeneralise descriptions of potential or capability. For instance, in Example 2, the statement of Beijing’s position does not express an attitude, yet the LLM labelled it as Capacity-Pos. In political contexts, maintaining a clear stance can be seen as a manifestation of political strength or influence, which the LLM may have inferred from the context and interpreted as Capacity.

**Example 2**:

From Beijing’s position, One China refers to the People’s Republic of China, and the foundation for the peaceful development of cross-strait relations is the 1992 Consensus, leaving no room for ambiguity of “one country, two interpretations”.

(LLM marked as Capacity-Pos)

Underinterpretation, another type of mislabelling, is reflected in lower rates of Recall. This phenomenon was particularly evident in the identification of Security, Inclination, Propriety, and Capacity. The detection of Security was impeded by their subtle expression, which often involves nuanced implications of latent risks, and thus demand a high level of contextual comprehension from the model. The recognition of Inclination was complicated by the need for the model to discern individuals’ preferences or intentions, which necessitated a profound understanding of psychological states. The Recall for Propriety was low due to its inherent complexity, as this kind of attitude requires the model to assess not only the text’s literal meaning but also the cultural and ethical dimensions that underpin social norms and behavioural standards. Similarly, the identification of Capacity was fraught with difficulty, as it involved not just the direct articulation of capabilities but more often the implicit assessment through the description of behaviours and events. This demanded that the model possess the ability to delve into the deeper and subtler meanings embedded within the text on the one hand, and the ability to understand social criteria for evaluating these meanings on the other hand. For instance, the statement in Example 3 exhibited an attitude of Propriety by advocating a position on an international issue based on perceived fairness and justice. The LLM’s failure to identify this attitude may stem from its limited understanding of cultural and ethical norms in the particular context, which is beyond the model’s training.

**Example 3**:

Chinese Foreign Ministry spokesperson Geng Shuang emphasised that the countermeasures taken by China are entirely necessary responses to the unwarranted suppression of Chinese media organisations in the US, and he stated that the causes and responsibilities for the current situation are not on the Chinese side.

(LLM did not mark)

In addition to the evaluation scores, it is remarkable that the LLM can perceive the polarity of attitudes from the overall context of the text, rather than solely based on literal text reading. In Example 4, there are several words that are generally considered positive, such as “ingenious”, “unprecedented”, “admire”, “learn”, etc. However, the author actually uses these words in a negative way to create a special ironic effect. Such ironic texts are difficult to identify in previous automatic marking projects because the external expression of irony is often opposite to the actual meaning expressed. Previous automatic marking projects, which rely more on the external form of expression, would therefore mislabel. However, the LLM can recognise the use of irony by understanding the overall context and thus make the correct markings.

**Example 4**:

But now, [Person’s name] has actually used it for keeping a mistress and marrying a concubine. Keeping a mistress also involves politics, which is quite ingenious, unprecedented, and can be sent to the Chinese History Museum for admiration and study by future generations.

(LLM marked as Propriety-Neg)

The evaluation has revealed the LLM’s excellent performance in discerning attitudes, with an overall accuracy of 0.95 and an F1 score of 0.8, effectively identifying attitudes across various categories. Its capability to recognise implicit attitudes demonstrated its advanced ability to detect subtleties in language, although there were instances of overinterpretation and underinterpretation. These cases usually involve subtle linguistic expressions and require contextual understanding of complex human behaviours, social norms, etc. However, at this stage, we are not able to find patterns as to when and which attitudes tend to be overinterpreted or underinterpreted. This points to a major challenge of applying LLMs in attitude analysis, and discourse analysis in general, namely, their lack of social knowledge, as such knowledge may not be always linguistically expressed, not to mention made available to LLMs. On the other hand, the robust capacity of the LLM in understanding and interpreting complex linguistic expressions is evident from its identification of sarcasms and the correct labelling of their polarity.

### Case study

After evaluating the performance of the LLM in the task of attitude identification, we conducted a preliminary analysis of the attitudes expressed by the *ODN* towards China, as identified by the LLM. As previously mentioned, the LLM identified 38,214 attitudes from 40,000 expressions, the distribution of which can be seen in Table 2.

**Table 2:** Distribution of attitudes.

Attitude	Positive	Negative	Total
Affect	18,872	8,460	27,332
Judgement	6,394	2,653	9,047
Appreciation	702	1,133	1,835
Total	25,968	12,246	38,214

In terms of attitude types, Affect was the most prevalent, accounting for 71.52%, while Judgement and Appreciation were less represented, at 23.67% and 4.80% respectively. The high proportion of Affect in the *ODN*’s coverage suggested a deliberate emphasis on emotional reactions in their reporting. By prioritising emotions, the newspaper was able to craft narratives that resonate more closely with the readers’ own experiences. This approach enhanced the relatability in the portrayal of China, thereby amplifying its appeal and emotional impact on the audience. In terms of polarity, positive attitudes accounted for 67.95%, with negative attitudes making up the remaining 32.05%. The predominance of positive attitudes indicated that the *ODN* adopted a more positive perspective in its reporting, aiming to shape a favourable perception of China. It provided empirical support for *ODN*’s pro-China stance, whose reports were aligned with the objectives of national image construction.

Beyond the general characteristics of attitudes, we also observed the complexity of attitudinal expression in the *ODN*, i.e., the presence of both singular-attitude and compound-attitude expressions. Specifically, there were 7,245 instances of attitude expressed independently, representing a singular-attitude expression. The remaining 30,969 instances were found within 8,955 expressions, constituting compound-attitude expressions. Among these singular-attitude expressions, Inclination was prominently featured, reflecting the *ODN*’s alignment with China’s stance and articulating China’s political inclinations. In the compound-attitude expressions, a pattern centred on Happiness was highlighted, which underscores the optimistic affective tone in the newspaper’s portrayal of China. This pattern demonstrates how, within individual expressions, Happiness extends to other attitudes, showcasing a micro-level multi-attitude expression pattern.

More specifically, there were 3,883 instances of the singular-attitudes of Inclination, representing 53.60% of all singular-attitude expressions. This was followed by Reaction and Security, which accounted for 10.64% and 9.12% respectively. Inclination was an important attitude in political discourse, reflecting the preferences, intentions, or trends of political actors and expressing their positions on specific issues or topics. The exclusive expression of singular-attitudes undoubtedly highlighted the significance of this attitude. Most of these Inclinations expressed by the *ODN* reflected China’s aspirations, such as promoting regional stability, fostering economic cooperation, and enhancing cultural exchanges. For instance, in Example 5, China expressed a positive expectation for India’s development and prosperity and a preference for India to play a more active role in international affairs, emphasising its commitment and importance through the act of “reiterating on multiple occasions”. Example 6 illustrates China’s adherence to an independent and peaceful foreign policy and a good-neighbourly and friendly regional diplomatic policy, reflecting its diplomatic inclinations. The statement also conveyed the desire to develop friendly relations with Asian countries, further demonstrating China’s proactive and constructive tendencies in regional relations. In these examples, China’s diplomatic discourse clearly expressed its inclinations regarding specific foreign policies and international relations development. This inclination not only reflected China’s diplomatic philosophy and values but also conveyed to the international community China’s intentions for interaction with other nations. Through such statements, the *ODN* played a role in shaping China’s international image and advancing China’s diplomatic agenda, which clearly reflected the newspaper’s adoption of a Chinese perspective.

**Example 5**:

China has repeatedly stated its hope to see a developed and prosperous India, one that plays a more active role in international affairs.

(LLM marked as Inclination-Pos)

**Example 6**:

China adheres to an independent and peaceful foreign policy and a good-neighbourly and friendly regional diplomatic policy, establishing and developing friendly relations with mutual understanding, trust, and cooperation with various Asian countries.

(LLM marked as Inclination-Pos)

The compound-attitude expressions encompass a multitude of attitudes within an individual expression, where these attitudes are not merely coexisting in isolation; instead, they interact in a manner that creates a coherent and dynamic flow of attitudes. In such an attitude flow, each attitude is a node, interconnected through implicit logical relationships and emotional nuances, forming an integration that collectively reflects the author’s comprehensive emotions and stance on a specific topic. The interdependent and flowing relationships within these attitude flows can reveal the deeper meanings of the text, as well as the underlying social, cultural, and psychological motivations. Table 3 presents the top 10 compound attitudes expressed by the *ODN* towards China in terms of frequency.

**Table 3:** Top 10 compound-attitudes expressed by *ODN* towards China and their frequencies.

Rank	Compound-attitude sequence	Freq.
1	capacity-positive→happiness-positive→normality-positive→satisfaction-positive→security-positive	602
2	happiness-positive→inclination-positive→satisfaction-positive→security-positive	525
3	happiness-positive→inclination-positive	272
4	inclination-positive→inclination-positive	260
5	capacity-positive→happiness-positive→satisfaction-positive→security-positive	253
6	capacity-negative→happiness-negative→normality-negative→satisfaction-negative→security-negative	246
7	happiness-positive→satisfaction-positive	232
8	happiness-positive→inclination-positive→normality-positive→satisfaction-positive→security-positive	221
9	happiness-positive→satisfaction-positive→security-positive	210
10	happiness-positive→inclination-positive→security-positive	204

Compound-attitudes are represented as a sequence of attitudes connected by logical relationships and emotional nuances.

The table reveals two key characteristics of the *ODN*’s portrayal of China through compound-attitudes. The first characteristic was the centrality of Happiness. Happiness not only frequently appeared on its own but also often co-occurred with other attitudes, collectively shaping the emotional tone of the text. The manifestation of Happiness can be broadly categorised into two patterns, which reveal how Happiness functions within different attitude combinations. In the first pattern, a positive emotional foundation was first established through Happiness, followed by further evaluation through other attitudes such as Inclination, Security, or Capacity. In the second pattern, Happiness emerged subsequent to other attitudes, such as Capacity, indicating that after the assessment of capability, which was the cause of Happiness, the author immediately expressed Happiness. Both patterns reflected the *ODN*’s strategy of conveying attitudinal messages through the combination of Happiness and other attitudes, yet they may differ in their focus, emotional intensity, and purpose. The first pattern was utilised to establish a sustained positive vibe, while the second pattern was employed to highlight various events and achievements and the author’s emotional reactions.

The second prominent feature was the logical chains of attitudes, in which different attitudes were intricately linked in a progressive manner. In Example 7, the narrative of China’s economy started with phrases such as “rapid development” and “a scale exceeding ten trillion yuan”, which set the stage for an attitude of awe and appreciation for the economic magnitude, marked as Capacity-Pos. This further led to an optimistic assessment of the beginning of the year, namely “[t]he start of this year was also very positive”, which expressed a sense of happiness and a positive attitude. Building on this positive sentiment, the text progressed to a more evaluative stance with the phrase “a more mature market economy has been formed”, which reflected a normalisation of the economic progress, indicating an expected state of affairs, thus Normality-Pos. The subsequent descriptions of economic growth and development further articulated a sense of Satisfaction with the economic performance. Finally, the author’s confidence in the economy’s ability to withstand potential shocks encapsulated a sense of Security or reassurance, which was identified as Security-Pos. Through the logical progression of the attitude chain, each attitude seamlessly transitioned into the next, creating a comprehensive and positive evaluative framework that reflected the author’s stance on China’s economic state. This incremental progression allowed readers to trace the development of attitudes from an initial observation of economic growth to a final expression of confidence in the economy’s resilience.

**Example 7**:

China’s economy has experienced rapid development over the past two decades, with a scale exceeding ten trillion yuan. The start of this year was also very positive, and notably, a more mature market economy has been formed. Although the economy may face some impact this year, it will not be too significant.

(LLM marked as Capacity-Pos, Happiness-Pos, Normality-Pos, Satisfaction-Pos, and Security-Pos)

In all, the *ODN* demonstrated a clear positive stance in its reporting on China. The singular-attitude expressions highlighted Inclination, reflecting the explicit adoption of a Chinese perspective. The compound-attitude expressions, with Happiness at its core, established an optimistic emotional tone, emphasising China’s positive role in international relations and its harmonious diplomatic intentions. Overall, the *ODN*’s coverage tended to emphasise China’s developmental achievements and its positive image on the international stage, presenting the positive impact of China’s rise and the normalcy of political changes through a logical chain of attitudes, thereby conveying a supportive stance towards China.

### Limitations and proposed solutions

LLMs have demonstrated the potential to surpass traditional NLP methods in capturing implicit attitudes. However, they still face certain limitations, particularly in their reliance on the quality of training data when identifying and analysing implicit attitudes. These models may struggle with discerning subtle linguistic cues and contextual implications, leading to inaccuracies in capturing nuanced attitudes and other meanings, manifested as overinterpretation or underinterpretation. Specifically, LLMs may erroneously interpret neutral statements as carrying implicit attitudes or fail to recognise the subtle attitudes implied in the text.

The emergence of these issues can be attributed to several factors. First, LLMs may lack necessary contextual information and therefore inadequate when dealing with complex situations that require an in-depth understanding of individual psychological states, cultural, and ethical dimensions, especially situations involving the subtleties of human psychology and specific social norms. This often leads to heightened sensitivity to textual cues or overgeneralised comprehension of contextual information. Second, LLMs may encounter difficulties in processing information in new domains, particularly in situations that demand specific domain knowledge, such as the language within the context of the Hong Kong press in this study. Finally, the factual knowledge embedded in LLMs may have a clear temporal boundary and lack the most up-to-date knowledge in specific domains, leading to inaccurate inferences when dealing with issues that require the latest domain knowledge. These limitations underscore the necessity for further training on diverse and specialised datasets. Given the rapid pace of generative AI development, it is safe to say that these issues can be resolved within the foreseeable future through continuous learning and the updating of models with the latest domain-specific information.

To address these challenges from the perspective of discourse analysis, the following directions are proposed. First, researchers can enhance the attitude recognition capabilities of LLMs by leveraging manually annotated data for fine-tuning, ensuring that models learn from accurate and reliable labels. The dataset should be diverse and balanced, encompassing a range of attitudinal expressions, including data that reflects various attitudinal scenarios, capturing the full spectrum of attitudes in terms of type and polarity. Incorporating data from multiple social and cultural contexts is essential for fine-tuning LLMs to accurately interpret attitudes shaped by diverse contextual factors. The dataset should also include a variety of rhetorical styles, as the use of rhetorical devices and linguistic techniques significantly influences the perception of attitudes within textual data. Models fine-tuned on such a diverse range of rhetorical expressions will be better equipped to handle the nuances of language in attitude recognition tasks. Secondly, prompt engineering is a crucial aspect in enhancing the capabilities of LLMs. It encompasses the careful crafting and rigorous testing of input prompts to effectively steer the model’s responses. During the enhancement of zero-shot attitude detection, researchers can bolster model performance by integrating expansive model knowledge and adopting thematic iterative data augmentation strategies. This approach acts as an adjunct to fine-tuning, allowing LLMs to render more precise inferences in the absence of explicit training data, leveraging the context and examples embedded within the prompts. In addition to fine-tuning and prompt engineering, post-processing steps are essential. These include error analysis and correction, feedback loop integration, and data augmentation. By implementing these steps, researchers can refine the model’s outputs to align more closely with the original intentions of the authors and the deeper implications of the text.

Another factor contributing to the struggle in attitude recognition by LLMs may lie within the attitude framework itself, for example, the complexity of the Capacity category. This category encompasses a range of semantic domains, such as “clever” and “rich”, which can vary significantly in meaning and context. The inherent complexity of such a category poses challenges for LLMs in accurately identifying and classifying attitudes, as it requires a nuanced understanding that goes beyond surface-level lexical matches. This insight suggests that the theoretical framework itself may need refinement to better accommodate the diverse and context-dependent nature of attitudinal expressions. The findings of this study, therefore, offer implications for the refinement of the attitude system, as well as other linguistic/discursive frameworks.

## Conclusion

This study introduces the application of LLMs for the automated analysis of media attitudes, marking a novel approach within the field of discourse analysis. By employing the Attitude system and a locally deployed Llama2 model, we conducted an in-depth analysis of the *ODN*’s reports on China. Our findings demonstrate that LLMs hold significant potential in identifying and classifying attitudes within texts, efficiently processing large volumes of textual data, and providing analysis results comparable to those of manual annotation.

Upon analysing the results identified by the LLM, we observed that *ODN*’s reports on China emphasised the attitude of Inclination in singular-attitude expressions to highlight China’s perspective, while compound-attitude expressions cantered around Happiness, establishing an optimistic emotional tone in the coverage. The reports tended to present a positive attitude, emphasising China’s developmental achievements and its positive image on the international stage. Moreover, the annotation process facilitated by the LLM enabled us to identify the existence of attitude chains in text, shedding light on the intricate relationships between attitudes.

Future research should explore the potential of various models (e.g., ChatGPT) and the strategies of fine-tuning to enhance LLMs’ attitude analysis capabilities across diverse cultural and linguistic contexts, as well as the integration of expert knowledge to improve the accuracy and depth of analysis. As generative AI technology advances, we anticipate that LLMs will play an increasingly pivotal role in discourse analysis, bringing both opportunities and challenges [32]. Based on this preliminary study, we may conclude that generative AI cannot replace human interpretation, but the results of LLM-based analysis may provide analysts with more empirical data to support their contextual and critical interpretations.
